# Macronutrient metabolism by the human gut microbiome: major fermentation by-products and their impact on host health

**DOI:** 10.1186/s40168-019-0704-8

**Published:** 2019-06-13

**Authors:** Kaitlyn Oliphant, Emma Allen-Vercoe

**Affiliations:** Department of Molecular and Cellular Biology, University of Guelph, 50 Stone Rd E, Guelph, ON N1G 2W1 Canada

**Keywords:** Human gut microbiome, Microbial metabolism, Macronutrients, Human health

## Abstract

The human gut microbiome is a critical component of digestion, breaking down complex carbohydrates, proteins, and to a lesser extent fats that reach the lower gastrointestinal tract. This process results in a multitude of microbial metabolites that can act both locally and systemically (after being absorbed into the bloodstream). The impact of these biochemicals on human health is complex, as both potentially beneficial and potentially toxic metabolites can be yielded from such microbial pathways, and in some cases, these effects are dependent upon the metabolite concentration or organ locality. The aim of this review is to summarize our current knowledge of how macronutrient metabolism by the gut microbiome influences human health. Metabolites to be discussed include short-chain fatty acids and alcohols (mainly yielded from monosaccharides); ammonia, branched-chain fatty acids, amines, sulfur compounds, phenols, and indoles (derived from amino acids); glycerol and choline derivatives (obtained from the breakdown of lipids); and tertiary cycling of carbon dioxide and hydrogen. Key microbial taxa and related disease states will be referred to in each case, and knowledge gaps that could contribute to our understanding of overall human wellness will be identified.

**Electronic supplementary material:**

The online version of this article (10.1186/s40168-019-0704-8) contains supplementary material, which is available to authorized users.

## Introduction

The human gut microbiota is a complex ecosystem of microorganisms that inhabits and critically maintains homeostasis of the gastrointestinal (GI) tract [1]. Most of the contributions made by the gut microbiota to the physiology of the human superorganism are related to microbial metabolism [2–4], with bacteria being the largest of these contributors to ecosystem functioning in terms of relative genetic content [2]. In general, microbial metabolism of both exogenous and endogenous substrates to nutrients useable by the host is the direct benefit, but metabolites can also act to modulate the immune system through impacting the physiology and gene expression of host cells [3, 5, 6]. The colon is the major site of this fermentation, as its relatively high transit time and pH coupled with low cell turnover and redox potential presents more favorable conditions for the proliferation of bacteria [7]. However, that does not preclude the importance of the microbiota at other sites, as for example, the small intestinal microbiota has been shown to regulate nutrient absorption and metabolism conducted by the host [8]. Further, the presence of diverse metabolic activity can allow the microbiota to maximally fill the available ecological niches and competitively inhibit colonization by pathogens at all sites [9–11]. The elevated concentrations of the mostly acidic fermentation by-products also locally reduce the pH to create a more inhospitable environment for these incoming invaders [11]. However, specific fermentation pathways carried out by gut microbes can result in the formation of toxic compounds that have the potential to damage the host epithelium and cause inflammation [12–14].

The three macronutrients consumed in the human diet, carbohydrates, proteins, and fat, can reach the colon upon either escaping primary digestion once the amount consumed exceeds the rate of digestion, or resisting primary digestion altogether due to the inherent structural complexity of specific biomolecules [14–16]. Several factors can influence digestive efficiency, which in turn modulates the substrates available to the gut microbiota for consumption, including the form and size of the food particles (affected by cooking and processing), the composition of the meal (affected by the relative ratios of macronutrients and presence of anti-nutrients such as α-amylase inhibitors), and transit time [17]. Transit time in particular has been shown to increase the richness and alter the composition of fecal microbial communities [18], which itself results from several variables including diet, physical activity, genetics, drugs (e.g., caffeine and alcohol), and psychological status [19]. The bioavailability of micronutrients to the host can also be influenced by gut microbial metabolic processes. Colonic bacteria can endogenously synthesize essential co-factors for host energy metabolism and regulation of gene expression, such as B vitamins [20]. Another example includes the biotransformation of exogenous plant-derived polyphenols that have anti-oxidant, anti-cancer, and/or anti-inflammatory properties by the gut microbiota, which improves their uptake by the host [21]. The following review articles on micronutrients are recommended to readers since this topic encompasses a wide scope of material [20, 21], as such, the predominant food sources that act as precursors for the most highly concentrated metabolites will be the focus of discussion here. The aim of this review is thus to describe the major microbial fermentation by-products derived from macronutrients and their subsequent impacts on host health.

## Primary degradation

Dietary polysaccharides can be interlinked in complex ways through a diverse array of bonds between monosaccharide units, reflected by the sheer number of carbohydrate-activating enzymes reported to have been found in the human gut microbiome [22]. For example, *Bacteroides thetaiotaomicron* possesses 260 glycoside hydrolases in its genome alone [23], which emphasizes the evolutionary requirement for adaptation in order to maximize utilization of resistant starch and the assortment of fibers available as part of the human diet. In contrast, human cells produce very few of these enzymes (although they do produce amylase to remove α-linked sugar units from starch and can use sugars such as glucose, fructose, sucrose, and lactose in the small intestine) and so rely on gut microbes to harvest energy from the remaining complex carbohydrates [17, 24]. However, once the rate-limiting step of primary degradation is surpassed, the resulting monosaccharides can be rapidly consumed by the gut microbiota with often little interconversion necessary for substrates to enter the Embden-Meyerhof-Parnas pathway, Entner-Doudoroff pathway, or Pentose phosphate pathway for pyruvate and subsequent ATP production [25]. Conversely, dietary proteins are characterized by conserved peptide bonds that can be broken down by proteases; gut bacteria can produce aspartic-, cysteine-, serine-, and metallo-proteases, but in a typical fecal sample, these bacterial enzymes are far outnumbered by proteases arising from human cells [26]. However, the 20 proteinogenic amino acid building blocks require more interconversion steps for incorporation into biochemical pathways in comparison to monosaccharide units, and thus it is not typical for a given gut microbial species to have the capacity to ferment all amino acids to produce energy [27]. Additionally, microbial incorporation of amino acids from the environment into anabolic processes would conserve more energy in comparison to their catabolic use, by relieving the necessity for amino acid biosynthesis [13]. It is for this reason that amino acids are generally not considered to be as efficient of an energy source as carbohydrates for human gut-associated microbes, and thus no surprise that the gut microbiota preferentially consume carbohydrates over proteins depending on the ratio presented to them [28, 29]. This metabolic hierarchy is analogous to human cells such as intestinal epithelial cells (IECs), in which increased amounts of autophagy occurs when access to microbially derived nutrients is scarce, as shown in germ-free mouse experiments [30]. However, there are notable exceptions to this general rule, as certain species of bacteria have adopted an asaccharolytic lifestyle, likely as a strategy to evade competition (examples included in Table 1).

**Table 1:** Major genera present in the human gut microbiome and their metabolisms

Phylum	Family	Genus	Substrates	Metabolism	End products
Actinobacteria	Bifidobacteriaceae	*Bifidobacterium*	Dietary carbohydrates HMO Mucin	Bifid shunt pathway	Acetate Ethanol Formate Lactate
Bacteroidetes	Bacteroidaceae	***Bacteroides***	Dietary carbohydrates HMO Mucin Proteins Succinate	1,2-Propanediol pathway^I^ Acetate production Ethanol production Succinate pathway	1,2-Propanediol Acetate Carbon dioxide and Hydrogen Ethanol Formate Propionate Succinate
	Porphyromonadaceae	***Parabacteroides*** ^W^	Dietary carbohydrates Proteins Succinate	Acetate production Succinate pathway	Acetate Carbon dioxide and Hydrogen Formate Propionate Succinate
	Prevotellaceae	***Prevotella*** ^NW^	Dietary carbohydrates Proteins Succinate	Acetate production Succinate pathway^I/A^	Acetate Formate Propionate Succinate
	Rikencellaceae	***Alistipes*** ^W^	Dietary carbohydrates Proteins Succinate	Acetate production Succinate pathway	Acetate Carbon dioxide and Hydrogen Formate Propionate Succinate
Firmicutes	**Clostridiaceae**	*Clostridium* (*Clostridium* cluster I)	Ethanol and Propionate Lactate Proteins Saccharides	1,2-Propanediol pathway^I^ Acetate production Acrylate pathway Butyrate kinase pathway Ethanol production Lactate production Valerate production	1,2-Propanediol Acetate Carbon dioxide and Hydrogen Ethanol Formate Lactate Propionate Butyrate Valerate
	Eubacteriaceae	*Eubacterium*	Acetate Carbon dioxide and hHydrogen Formate Lactate Methanol Proteins Saccharides	Acetogenesis Acetate production Butyryl c CoA transferase pathway Ethanol production Lactate production	Acetate Butyrate Carbon dioxide and Hydrogen Ethanol Formate Lactate
	**Erysipelotrichaceae**	*Erysipelatoclostridium*	Proteins Saccharides	Acetate production Lactate production	Acetate Carbon dioxide and Hydrogen Formate Lactate
	**Lachnospiraceae**	***Blautia*** (*Clostridium* cluster XIVa)	1,2-Propanediol Carbon dioxide and Hydrogen Dietary carbohydrates Formate Mucin	1,2-Propanediol pathway Acetogenesis Acetate production Ethanol production Lactate production Succinate pathway^I^	Acetate Carbon dioxide and Hydrogen Ethanol Formate Lactate Propanol Propionate Succinate
		***Coprococcus*** (*Clostridium* cluster XIVa)	Acetate Dietary carbohydrates Lactate	Acrylate pathway Butyrate kinase pathway Butyryl CoA:acetyl CoA transferase pathway Ethanol production Lactate production	Acetate Butyrate Ethanol Carbon dioxide and Hydrogen Formate Lactate Propionate
		***Dorea*** (*Clostridium* cluster XIVa)	Dietary carbohydrates	Acetate production Ethanol production Lactate production	Acetate Carbon dioxide and Hydrogen Ethanol Formate Lactate
		***Lachnoclostridium*** (*Clostridium* cluster XIVa)	Proteins Saccharides	Acetate production Butyrate kinase pathway Ethanol production Lactate production	Acetate Butyrate Carbon dioxide and Hydrogen Ethanol Formate Lactate
		***Roseburia*** (*Clostridium* cluster XIVa)	1,2-Propanediol Acetate Dietary carbohydrates	1,2-Propanediol pathway Acetate production Butyryl CoA:acetyl CoA transferase pathway Ethanol production Lactate production	Acetate Butyrate Carbon dioxide and Hydrogen Ethanol Formate Lactate Propanol Propionate
	Lactobacillaceae	*Lactobacillus*	1,2-Propanediol Saccharides	1,2-Propanediol pathway Acetate production Ethanol production Lactate production	Acetate Ethanol Formate Lactate Propanol Propionate
	**Ruminococcaceae**	***Faecalibacterium*** (*Clostridium* cluster IV)	Acetate	Butyryl CoA:acetyl CoA transferase pathway	Butyrate Carbon dioxide and Hydrogen Formate
		***Ruminiclostridium*** ^W^ (Specifically *Clostridium* cluster IV, which is currently grouped with *Clostridium* cluster III)	Dietary carbohydrates Proteins	Acetate production Butyrate kinase pathway Ethanol production Lactate production	Acetate Butyrate Carbon dioxide and Hydrogen Ethanol Formate Lactate
		*Ruminococcus* (*Clostridium* cluster IV)	Dietary carbohydrates	Acetate production Ethanol production Lactate production Succinate pathway^I^	Acetate Ethanol Formate Lactate Succinate
	Streptococcaceae	***Streptococcus*** ^NW^	Mucin Saccharides	Acetate production Ethanol production Lactate production	Acetate Ethanol Formate Lactate
	**Veillonellaceae**	*Veillonella*	1,2-Propanediol Lactate Proteins Saccharides Succinate	1,2-Propanediol pathway Acetate production Lactate production Succinate pathway	Acetate Carbon dioxide and Hydrogen Formate Lactate Propanol Propionate Succinate
Proteobacteria	Enterobacteriaceae	*Escherichia*	Proteins Saccharides	1,2-Propanediol pathway^I^ 2,3-Butanediol production Acetate production Ethanol production Lactate production Succinate pathway^I^	1,2-Propanediol 2,3-Butanediol Acetate Carbon dioxide and Hydrogen Ethanol Formate Lactate Succinate

Taxa that are listed as part of a ‘core’ gut microbiota found by Falony et al. are in bold [31]. Those genera that were core components of exclusively the ‘Western’ cohorts are denoted with a ‘W’ superscript, whereas the exclusively ‘non-Western’ ones are denoted with a ‘NW’ superscript. If the core taxon could not be resolved to the genus level, the bacterial families are bolded. For the bacterial families that do not already contain several core genera, the most commonly described genus of the human gut microbiome for that family is also listed as a representative. Additionally, genera found to be highly prevalent among the human population, yet typically present in low abundance, are underlined [32]. The possible substrates consumed, metabolisms, and metabolites for each genus are listed. These metabolisms were inferred from the following articles [28, 33–61]. Note that many of these metabolisms are species-specific, and only the substrates commonly utilized among species of the genus are listed. Further, only the most abundant metabolites produced from pyruvate catabolism (i.e., saccharolytic processes) are given. When a particular metabolic pathway is denoted with an ‘I’ superscript, the microorganisms do not possess the full enzymatic pathway, but rather produce the typical intermediate as an end-product instead. Likewise, an ‘I/A’ indicates species of that genus may possess either the full or half pathway

## Pyruvate metabolism

**Fig. 1:**
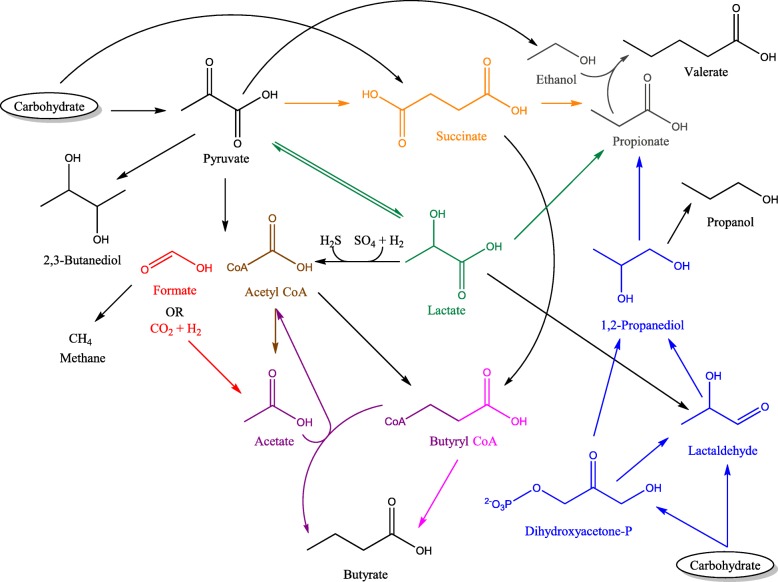
Strategies of pyruvate catabolism by the human gut microbiome. Carbohydrates are first degraded to pyruvate. Pyruvate may then be converted to succinate, lactate, acetyl CoA + formate/carbon dioxide + hydrogen, ethanol, or 2,3-butanediol. Succinate may, however, also be a direct product of carbohydrate fermentation. Succinate and lactate do not typically reach high concentrations in fecal samples, as they can be further catabolized to produce energy, but certain species do secrete them as their final fermentation end-product, which enables cross-feeding. Acetate is produced by two pathways; (1) through direct conversion of acetyl CoA for the generation of energy (brown) or (2) acetogenesis (red). Formate/carbon dioxide + hydrogen can also be substrates for methanogenesis. Propionate is produced by three pathways; (1) the succinate pathway (orange), (2) the acrylate pathway (green), or (3) the 1,2-propanediol pathway (blue). 1,2-Propanediol is synthesized from lactaldehyde or dihydroxyacetone phosphate, which both are products of deoxy sugar fermentation (e.g., fucose, rhamnose). Alternatively, lactaldehyde can be produced from lactate, or 1,2-propanediol can be fermented to propanol. Propionate can be coupled with ethanol for fermentation to valerate (gray). The precursor for butyrate, butyryl CoA, is generated from either acetyl CoA or succinate. Butyrate is then produced by two pathways; (1) the butyrate kinase pathway (pink) or (2) the butyryl CoA:acetyl CoA transferase pathway (purple). Butyrate-producing bacteria may also cross-feed on lactate, converting it back to pyruvate. Lactate may also be catabolized as part of sulfate reduction

Once pyruvate is produced, primarily from carbohydrates but also from other substrates, the human gut microbiota has developed several fermentation strategies to further generate energy, which are depicted in Fig. 1. Pyruvate can either be catabolized into succinate, lactate, or acetyl-CoA. However, these intermediates do not reach high concentrations in typical fecal samples, as they can be further metabolized by cross-feeders, producing the short-chain fatty acids (SCFAs) acetate, propionate, and butyrate (Table 1) [33]. These fecal metabolites are the most abundant and well-studied microbial end-products, since their effects are physiologically important: for example, host intestinal epithelial cells (IECs) utilize them as a source of fuel [62]. Indeed, SCFAs contribute approximately 10% of the caloric content required by the human body for optimal functioning [63]. Butyrate is the most preferred source of energy in this respect; its consumption improves the integrity of IECs by promoting tight junctions, cell proliferation, and increasing mucin production by Goblet cells [63, 64]. Butyrate also exhibits anti-inflammatory effects, through stimulating both IECs and antigen presenting cells (APCs) to produce the cytokines TGF-β, IL-10, and IL-18, and inducing the differentiation of naïve T cells to T regulatory cells [65]. Acetate and propionate can also be consumed by IECs (though to a much lesser degree than butyrate) and have some anti-inflammatory effects [33, 63]. Both acetate and propionate can dampen pro-inflammatory cytokine production mediated by toll-like receptor (TLR) 4 stimulation, and propionate, similar to butyrate, can induce the differentiation of T cells to T regulatory cells [33, 34]. Excess SCFAs that are not metabolized by IECs are transported via the hepatic vein to the liver, where they can be incorporated as precursors into gluconeogenesis, lipogenesis, and cholesterologenesis [62]. Specifically, propionate is gluconeogenic, whereas acetate and butyrate are lipogenic. The ratio of propionate to acetate is thought to be particularly important, as propionate can inhibit the conversion of acetate to cholesterol and fat [62, 66]. Indeed, propionate administration alone can reduce intra-abdominal tissue accretion and intrahepatocellular lipid content in overweight adults [67]. The role(s) of SCFAs in glucose homeostasis is/are not yet fully elucidated, although preliminary work has additionally suggested a beneficial effect, since plasma insulin levels are inversely related to serum acetate concentrations [62, 68].

In addition to SCFAs, small but significant amounts of alcohols, including ethanol, propanol, and 2,3-butanediol, can be formed as end-products of pyruvate fermentation (Table 1; Fig. 1). A further alcohol, methanol, is also produced by the gut microbiota as a result of pectin degradation, demethylation of endogenous cellular proteins for regulation, or vitamin B_12_ synthesis [69] rather than fermentation. Alcohols are transported to the liver, where the detoxification process involves their conversion to SCFAs, although through pathways that yield toxic aldehydes as precursors [69–71]. Higher concentrations of endogenous alcohols are thus thought to be a contributing factor to the development of non-alcoholic fatty liver disease (NAFLD) [70, 72]. Proteobacteria are known to be particularly capable of alcohol generation [69, 72], and are, interestingly, positively associated with dysbiosis in inflammatory bowel disease (IBD) [73], a disease in which patients are predisposed to developing NAFLD [74]. However, alcohols can also be detoxified by many members of the gut microbiota via pathways similar to those present in mammalian cells, regulating their concentration [69]. Additionally, methanol can be used as a substrate for methanogenesis or acetogenesis [35, 69, 75], and ethanol can be coupled to propionate for fermentation to the SCFA, valerate (Table 1) [36]. Valerate is a poorly studied metabolite, but it has been shown to inhibit growth of cancerous cells [76] and to prevent vegetative growth of *Clostridioides difficile* both in vitro and in vivo [36].

## Hydrogenotrophy

The human body may rapidly absorb SCFAs and alcohols, which helps to reduce their nascent concentrations within the colon, allowing for continued favorable reaction kinetics [15, 77] . In addition, the gaseous fermentation by-products, carbon dioxide and hydrogen, must also be removed to help drive metabolism forward. The utilization of these substrates is mainly the result of cross-feeding between gut microbiota members, rather than host absorption. Three main strategies for this activity exist in the human gut: (1) acetogens, for example, *Blautia* spp., convert carbon dioxide plus hydrogen to acetate (further examples included in Table 1); (2) methanogens, namely archaea such as *Methanobrevibacter*, convert carbon dioxide plus hydrogen to methane; and (3) sulfate reducing bacteria, including *Desulfovibrio*, convert sulfate plus hydrogen to hydrogen sulfide [15, 37]. A higher abundance of these cross-feeders may improve the overall efficiency of metabolism in the gut; for example, an increase in methanogens is observed in the GI tract of anorexia nervosa patients, which may be a coping strategy by the gut microbiota in response to a lack of food sources [78, 79]. Sulfate-reducing bacteria are the most efficient of the hydrogenotrophs, but require a source of sulfate; in the gut, the most prominent source of sulfate is sulfated glycans [80]. Although some of these glycans may be obtained from the diet, the most accessible source is mucin produced by the host [38]. Sulfate-reducing bacteria obtain sulfate from these substrates via cross-feeding with microbes such as *Bacteroides*, which produce sulfatases [80, 81]*.* Hydrogen sulfide is both directly toxic to IECs through inhibition of mitochondrial cytochrome C oxidase, and pro-inflammatory via activation of T helper 17 cells [82, 83]. Hydrogen sulfide can additionally directly act on disulfide bonds in mucin to further facilitate mucin degradation [84]. Elevated hydrogen sulfide concentrations and increased proportions of sulfate-reducing bacteria are reported in IBD [85].

## Catabolism of amino acids

The digestibility of proteins by the host is more variable than that of carbohydrates and fats, and is influenced by the previously mentioned factors of food processing, macronutrient ratios, and transit time [14, 18], in addition to its source (e.g., plant or animal), which also leads to different amino acid compositions available to the gut microbiota [14, 86]. The extra steps of interconversion required for amino acid fermentation yield a large diversity of by-products. Protein catabolism in the gut generally has a negative connotation, as compounds that are toxic to the host can result from this process, including amines, phenols/indoles, and sulfurous compounds [12–14]. However, it is important to note that not all amino acids are fermented to toxic products as a result of gut microbial activity; in fact, the most abundant end products are SCFAs [13, 14]. Therefore, it may not be protein catabolism per se that negatively impacts the host, but instead specific metabolisms or overall increased protein fermentation activity. It is thus important to examine these subtleties. A microbe can exhibit one of two strategies for the initial step of amino acid catabolism, either deamination to produce a carboxylic acid plus ammonia or decarboxylation to produce an amine plus carbon dioxide [12]. Ammonia can inhibit mitochondrial oxygen consumption and decrease SCFA catabolism by IECs, which has led to the assumption that excess ammonia production can negatively impact the host [87–89]. However, the gut microbiota also rapidly assimilates ammonia into microbial amino acid biosynthetic processes [13], and host IECs can additionally control ammonia concentration through conversion to citrulline and glutamine, or through slow release into the bloodstream [90, 91]. It is thus unclear how much protein catabolism is necessary to achieve toxic ammonia concentrations, and this may vary between hosts. This uncertainty, coupled with the multiple negative impacts amines can have on the host (discussed below), have led to speculation that deamination would improve host outcomes. Fortunately, deamination appears to be the more common strategy of amino acid catabolism by the gut microbiota, because high concentrations of SCFAs are produced from amino acid degradation via this pathway [12, 13]. The next steps depend on the class of amino acid starting substrate, with most eventually resulting in tricarboxylic acid cycle intermediates, pyruvate, or coenzyme A-linked SCFA precursors [39, 75]. An exception would be the series of Stickland reactions exhibited by certain Clostridia*,* in which a coupled oxidation and reduction of two amino acids occurs as an alternative to using hydrogen ions as the electron acceptor [40, 41]. Phosphate is simultaneously added to the reduced amino acid in this case, and thus oxidative phosphorylation for the production of ATP can occur directly from the resultant acyl phosphate. In turn, branched-chain fatty acids (BCFAs), such as isovalerate and isobutyrate, can be produced as end-products. Additionally, some gut microbial species, mainly from the class Bacilli, also possess a specialized branched-chain keto acid dehydrogenase complex to yield energy from the oxidized forms of the branched-chain amino acids directly, which also leads to BCFA production [13, 75]. The major SCFA and BCFA products generated from degradation of each amino acid are presented in Table 2. BCFAs are often used as a biomarker of protein catabolism, with the promoted goal to reduce their concentration in order to improve health outcomes [14]. However, little is actually known about the impact of BCFAs on host health. In fact, preliminary work has shown that BCFAs are able to modulate glucose and lipid metabolism in the liver similarly to SCFAs [93], and isobutyrate can be used as a fuel source by IECs when butyrate is scarce [94]. What is undisputed, however, are the negative consequences of the pro-inflammatory, cytotoxic, and neuroactive compounds yielded from the sulfur-containing, basic and aromatic amino acids.

**Table 2:** Major products of amino acid fermentation by the human gut microbiota

Amino acid	Amino acid class	Major products
Aspartate	Acidic	Propionate
Glutamate	Acidic	Acetate, Butyrate
Alanine	Aliphatic	Acetate, Propionate, Butyrate
Glycine	Aliphatic	Acetate Methylamine
Isoleucine	Aliphatic	2-Methylbutyrate or converted to Valine
Leucine	Aliphatic	Isovalerate
Proline	Aliphatic	Acetate
Valine	Aliphatic	Isobutyrate
Asparagine	Amidic	Converted to aspartate
Glutamine	Amidic	Converted to glutamate
Phenylalanine	Aromatic	Phenolic SCFA Phenylethylamine
Tryptophan	Aromatic	Indolic SCFA Tryptamine
Tyrosine	Aromatic	4-Hydroxyphenolic SCFA Tyramine
Arginine	Basic	Converted to other amino acids (mainly Ornithine) Agmatine
Histidine	Basic	Acetate, Butyrate Histamine
Lysine	Basic	Acetate, Butyrate Cadaverine
Serine	Hydroxylic	Butyrate
Threonine	Hydroxylic	Acetate, Propionate, Butyrate
Cysteine	Sulfur-containing	Acetate, Butyrate, Hydrogen sulfide
Methionine	Sulfur-containing	Propionate, Butyrate, Methanethiol

Listed are the compounds found to be above 1 mM concentration in in vitro fermentation experiments conducted by Smith and Macfarlane [92], in addition to the biogenic amines that can be produced by decarboxylation [12, 13]. Underlined are the products indicated as most abundant as reported in a review article by Fan et al. [12]

### Sulfur-containing amino acids

Catabolism of the sulfur-containing amino acids, cysteine and methionine, results in the production of hydrogen sulfide and methanethiol, respectively [13, 14], and a large number of taxonomically diverse bacterial species contain the requisite degradative enzymes within their genomes, including members of the Proteobacteria phylum, the Bacilli class, and the *Clostridium* and *Bifidobacterium* genera [13, 75]. Hydrogen sulfide can be methylated to methanethiol, which can be further methylated to dimethyl sulfide, and this methylation is thought to be part of the detoxification process due to the progressively less toxic nature of these compounds [95]. However, methanethiol may also be converted to hydrogen sulfide, then oxidized to sulfate, for detoxification; this sulfate can then be utilized by sulfate-reducing bacteria [80, 81, 95]. Indeed, this latter reaction has been observed in cecal tissue, and is part of the sulfur cycle of the gut [96]. The impact of hydrogen sulfide on host health has already been discussed, thus the focus will shift to the biogenic amines produced by basic amino acid fermentation and the phenol/indole compounds produced by aromatic amino acid fermentation.

### Basic amino acids

A wide diversity of bacterial species within the gut microbiota can decarboxylate the basic amino acids, thus resulting in the formation of amine by-products shown in Additional file 1, including bifidobacteria, clostridia*,* lactobacilli, enterococci, streptococci, and members of the Enterobacteriaceae family [97]*.* The catabolism of arginine can produce agmatine by deamination, and/or putrescine, spermidine, and spermine as part of the polyamine synthesis pathway (Additional file 1). Agmatine inhibits the proliferation of IECs, which is thought to stem from its ability to reduce the synthesis and promote the degradation of other polyamines [98]. This effect may not be negative depending on the context; for example, the resultant decrease of fatty acid metabolism in tissues reduced both weight gain and the hormonal derangements associated with obesity in rats fed a high fat chow [99]. Agmatine also may be anti-inflammatory through inhibition of nitric oxide synthase [100], and is a candidate neurotransmitter, with agonism for α_2_-adenoceptors and imidazoline binding sites, while simultaneously blocking ligand-gated cation channels (NMDA class) [101]. The latter activity has therapeutic potential for remediating some forms of hyperalgesia and for its neuroprotectivity. Putrescine, on the other hand, is essential for the proliferation of IECs [102]. It is the precursor to spermidine/spermine, which are both able to relieve oxidative stress and promote cellular longevity through autophagy stimulation [103]. All three polyamines improve the integrity of the gut by increasing expression of tight junction proteins [104], promoting intestinal restitution [105] and increasing mucus secretion [105, 106]. Finally, both putrescine and spermine are able to inhibit the production of pro-inflammatory cytokines, such as IL-1, IL-6, and TNF-α [107, 108]. Therefore, any benefits of agmatine must be weighed against its consequent reduction of these polyamines; it may be effective in the treatment of certain conditions such as metabolic syndrome but could be detrimental in excess under normal conditions. Arginine can additionally be converted to glutamate, which can be deaminated to produce 4-aminobutryate (GABA). GABA is the major inhibitory neurotransmitter of the central nervous system, and alterations in the expression of its receptor have been linked to the pathogenesis of depression and anxiety [109]. Administration of lactobacilli and bifidobacteria that produce GABA to mice and rats has resulted in a decrease of depressive behaviors, a reduction of corticosterone induced stress and anxiety, and lessened visceral pain sensation [109–111]. GABA can additionally regulate the proliferation of T cells and thus has immunomodulatory properties [112]. Interestingly, chronic GI inflammation not only induces anxiety in mice, but depression and anxiety often present comorbidity with GI disorders, including irritable bowel syndrome (IBS) [109, 113].

The catabolism of histidine can produce histamine (Additional file 1). Histamine may be synonymous with its exertion of inflammation in allergic responses, but bacterially produced histamine has actually been shown to inhibit the production of the pro-inflammatory cytokines TNF-α in vivo [114], and IL-1, and IL-12 in vitro [115], while simultaneously preventing intestinal bacterial translocation. Histamine is also a neurotransmitter, modulating several processes such as wakefulness, motor control, dendritic cell activity, pain perception, and learning and memory [116]. Low levels of histamine are associated with Alzheimer’s disease, convulsions, and seizures, and increasing its concentration has antinociceptive properties [117]. However, there is likely a range of suitable concentration, as high levels of histamine are associated with sleep disorders, Parkinson’s disease, schizophrenia, and autism [116, 117].

The catabolism of lysine can produce cadaverine (Additional file 1). Cadaverine is a poorly studied metabolite; it can be toxic, but only in high amounts [13, 97]. Cadaverine has, however, been shown to potentiate histamine toxicity [118] and higher concentrations of cadaverine are associated with ulcerative colitis (UC) [119].

### Aromatic amino acids

Aromatic amino acid degradation can yield a wide diversity of indolic and phenolic compounds that can act as toxins or neurotransmitters as shown in Additional file 2. The catabolism of tryptophan can produce tryptamine and indoles (Additional file 2). Tryptamine is a neurotransmitter that plays a role in regulating intestinal motility and immune function [120]. Particularly, it is able to interact with both indoleamine 2,3-dioxygenase and the aryl hydrocarbon receptor to heighten immune surveillance, and dampen the expression of pro-inflammatory cytokines, respectively [121, 122]. A lack of these activities has therefore been implicated in the pathology of IBD; although, it should be noted that most tryptophan metabolites can interact with these receptors, thus it is not tryptamine-specific [13, 120, 122]. Tryptamine can also both potentiate the inhibitory response of cells to serotonin and induce its release from enteroendocrine cells [120, 123]. Serotonin is a neurotransmitter involved in many processes including mood, appetite, hemostasis, immunity, and bone development [13, 124]. Its dysregulation is thus reported in many disorders, including IBD [125], IBS [126], cardiovascular disease [127], and osteoporosis [128]. Tryptophan decarboxylation is a rare activity among species of the gut microbiota, but certain Firmicutes have been found to be capable of it, including the IBD-associated species, *Ruminococcus gnavus* [129, 130]*.* Indole, on the other hand, is a major bacterial metabolite of tryptophan, produced by many species of *Bacteroides* and Enterobacteriaceae [120]*.* It plays an important role in host defense, by interacting with the pregnane X receptor and the aryl hydrocarbon receptor [120]. This activity fortifies the intestinal barrier by increasing tight junction protein expression and downregulates the expression of pro-inflammatory cytokines [120, 131]. It also induces glucagon like peptide-1 (an incretin) secretion by enteroendocrine cells, inhibiting gastric secretion and motility, to promote satiety [132, 133]. Indole is additionally a signaling molecule for bacteria, influencing motility, biofilm formation, antibiotic resistance, and virulence, and shown to inhibit the colonization capabilities of pathogens such as *Salmonella enterica* [134]. However, indole overproduction can increase its export to the liver, where it is sulfated to indoxyl sulfate, a uremic toxin associated with chronic kidney disease [135]. Further, its effects as a signaling molecule for both enteroendocrine cells and bacteria are dose dependent, with high concentrations rendering it ineffective [120, 132, 134]. Other indole metabolites are additionally able to interact with the pregnane X receptor and/or aryl hydrocarbon receptor in a similar fashion, thus benefiting the host, but are less well studied [120].

The catabolism of tyrosine can produce tyramine, phenols, and p-coumarate (Additional file 2). Tyramine is a neurotransmitter that can be produced by certain gut bacteria via decarboxylation, including *Enterococcus* and Enterobacteriaceae [97]. It is infamous for causing the ‘cheese reaction’ hypertensive crisis in individuals taking monoamine inhibitor class drugs, although it can additionally cause migraines and hypertension in sensitive individuals or a mild rise in blood pressure when consumed in excess by the general populace [136]. Tyramine facilitates the release of norepinephrine that induces peripheral vasoconstriction, elevates blood glucose levels, and increases cardiac output and respiration [137]. It has also been shown to increase the synthesis of serotonin by enteroendocrine cells in the gut, elevating its release into circulation [124]. Phenol and p-cresol are phenolic metabolites that have been shown to both decrease the integrity of the gut epithelium and the viability of IECs [138, 139], and can be produced by many gut bacterial species, such as members of the Enterobacteriaceae and *Clostridium* clusters I, XI, and XIVa [140]. P-cresol in particular is genotoxic, elevates the production of superoxide, and inhibits proliferation of IECs [141]. P-cresol may additionally be sulfated to cresyl sulfate in the gut or liver, which has been found to suppress the T helper 1-mediated immune response in mice [142], and, interestingly, phenolic sulfation was found to be impaired in the gut mucosa of UC patients [143]. Indeed, the colonic damage induced by unconjugated phenols is similar to that observed in IBD [138]. Cresyl sulfate is also associated with chronic kidney disease, however, as it can damage renal tubular cells through induction of oxidative stress [144]. This compound is also particularly elevated in the urine of autistic patients, but a causative link in this case has not been elucidated [145].

The catabolism of phenylalanine can produce phenylethylamine and trans-cinnamic acid (Additional file 2). Unlike tyrosine and tryptophan, little is known about these phenylalanine-derived metabolites. Phenylethylamine is a neurotransmitter that functions as an ‘endogenous amphetamine’ yielded from decarboxylation [136]. Through facilitating the release of catecholamine and serotonin, phenylethylamine in turn elevates mood, energy, and attention [146]. However, it has been reported that ingesting phenylethylamine can induce headache, dizziness, and discomfort in individuals with a reduced ability to convert it to phenylacetate, suggesting excessive amounts have negative consequences [136]. In terms of its production in the gut, phenylethylamine has thus been positively associated with Crohn’s disease and negatively correlated with *Faecalibacterium prausnitzii* in one study [147]. The conversion of phenylalanine to trans-cinnamate and tyrosine to p-coumaric acid results in increased phenylpropionate and 4-hydroxyphenylpropionate concentrations, which in turn produce urinary metabolites associated with the ‘chlorogenic acid’ phenotype in rats, as suggested by Clayton [148]. These metabolic pathways were found to so far specifically occur within species of *Clostridium* and *Peptostreptococcus*, respectively [149, 150]. The chlorogenic acid phenotype is associated with both autism and schizophrenia, suggesting a role of altered aromatic amino acid metabolism in these disorders [148, 151, 152]. However, further research is still needed, as there remains no mechanistic explanation of these metabolites toward disease development. Further, both trans-cinnamic acid and p-coumaric acid are negatively associated with cardiovascular disease [153, 154]. P-coumaric acid, in particular, is a common phenolic compound derived from plant matter that has anti-inflammatory properties, and has been demonstrated to prevent platelet aggregation [155]. Thus, these metabolites may simply be an indicator of altered microbial metabolism in general, when found in excess.

## Catabolism of lipids

A very small proportion of total dietary fat reaches the colon (< 5%) [16, 156]. Microorganisms in the gut are known to possess lipases, which can degrade triglycerides and phospholipids into their polar head groups and free lipids [16, 157]. Triglycerides represent 95% of total dietary fat, whereas phospholipids, mostly in the form of phosphotidylcholine, constitute a minor portion, but are also derived endogenously from bile acids [158]. Certain bacteria inhabiting the GI tract, including species of lactobacilli, enterococci, clostridia, and Proteobacteria, can utilize the backbone of triglycerides as an electron sink, reducing glycerol to 1,3-propanediol [159]. 3-Hydroxypropanal (reuterin) is an intermediate of this process that has been reported to accumulate extracellularly in cultures of *Lactobacillus* and *Enterococcus* spp*.* [160]*.* Reuterin has antimicrobial properties acting against pathogens and commensals alike [161], but it can also be spontaneously dehydrated to acrolein [71]. Acrolein is a highly reactive genotoxin, with an equivalent mutagenic potency to formaldehyde, raising concerns about this metabolic process [71, 159]. Meanwhile, choline can additionally be metabolized to trimethylamine by species of the gut microbiota, particularly Clostridia (especially members of *Clostridium* cluster XIVa and *Eubacterium* spp*.*) and Proteobacteria [162, 163]. Trimethylamine is oxidized in the liver to trimethylamine N-oxide [163, 164], which exacerbates atherosclerosis by promoting the formation of foam cells (lipid-laden macrophages) [164] and altering cholesterol transport [165]. High levels of serum trimethylamine N-oxide are thus associated with cardiovascular disease [166] and atherosclerosis [167]. However, it should be noted that active research in these areas is in its early stages, and thus the link between the gut microbiota-mediated lipid head group metabolism and health consequences is still unclear. For example, a study on the metabolism of glycerol by fecal microbial communities found that only a subset could reduce it to 1,3-propanediol, and the authors did not detect any reuterin [159]. Further, some members of the gut microbiota (e.g., methylotrophs) can breakdown trimethylamine to dimethylamine, so the actual amount of trimethylamine that is available for transportation to the liver can be diverted, and this is likely to be influenced by inter-individual variability in the composition of the gut microbiota [168].

In contrast to the polar head groups, microorganisms are not thought to have the ability to catabolize free lipids in the anaerobic environment of the gut [169]. However, free lipids have antimicrobial properties [169, 170] and can directly interact with host pattern recognition receptors. Particularly, saturated fatty acids are TLR4 agonists that promote inflammation [171], whereas omega-3 unsaturated fatty acids are TLR4 antagonists that prevent inflammation [172]. Interestingly, chronic inflammation co-occurring with obesity has been well described [173], and could be a result of the aforementioned pro-inflammatory properties of free lipids, the lack of anti-inflammatory SCFAs produced from carbohydrate fermentation (high-fat diets tend to be low in carbohydrates), or a combination of both. High-fat diets do have a reported impact on the composition of the gut microbiota, yet it is unclear whether it is the increased fat content per se or the relative decrease in carbohydrates, which often accompanies these diets, that is the chief influencer [16, 169]. Indeed, Morales et al. observed that a high-fat diet including fiber supplementation induces inflammation without altering the composition of the gut microbiota [16]. Regardless, the gut microbiota is required for the development of obesity, as shown in GF mice experiments, because of the ability of SCFAs to alter energy balance as previously discussed [174].

## Effect on endogenous substrate utilization

Metabolism of exogenous substrates greatly affects the use of endogenous substrates by the gut microbiota. Dietary fiber reduces the degradation of mucin, and the utilization of mucin is thought to cycle daily depending on the availability of food sources [175, 176]. Mucin is a sulfated glycoprotein [38], thus the same concepts of carbohydrate and protein degradation from dietary sources discussed above apply. However, it should be noted that mucin turnover by the gut microbiota is a naturally occurring process, and only when it occurs in elevated amounts does it have negative connotations. For example, *Akkermansia muciniphila* is a mucin-utilizing specialist that is depleted in the GI tract of IBD [177] and metabolic syndrome [178] patients. *A*. *muciniphila* has a demonstrated ability to cross-talk with host cells, promoting an increase in concentration of glucagon-like peptides, 2-arabinoglycerol, and antimicrobial peptides that improve barrier function, reduce inflammation, and induce proliferation of IECs [179]. Through this communication, *A*. *muciniphila* also, paradoxically, restored the thickness of the mucin layer in obese mice. Dietary fat intake can also alter the profile of bile acids. Dairy-derived saturated lipids increase the relative amount of taurine-conjugation, and this sulfur-containing compound leads to the expansion of sulfate-reducing bacteria in the gut [180]. Bile acid turnover is, however, a naturally occurring process, which modulates bile acid reabsorption, inflammation, triglyceride control, and glucose homeostasis from IEC signaling [181].

## Conclusions

The critical contributions of the gut microbiota toward human digestion have just begun to be elucidated. Particularly, more recent research is revealing how the impacts of microbial metabolism extend beyond the GI tract, denoting the so-called gut-brain (e.g., biogenic amines acting as neurotransmitters) [182], gut-liver (e.g., alcohols) [183], gut-kidney (e.g., uremic toxins such as cresyl sulfate) [135], and gut-heart (e.g., trimethylamine) [184] axes. The primary focus to date has been on the SCFAs derived mainly from complex carbohydrates, and crucial knowledge gaps still remain in this area, specifically on how the SCFAs modulate glucose metabolism and fat deposition upon reaching the liver. However, the degradation of proteins and fats are comparatively less well understood. Due to both the diversity of metabolites that can be yielded and the complexity of microbial pathways, which can act as a self-regulating system that removes toxic by-products, it is not merely a matter of such processes effecting health positively or negatively, but rather how they are balanced. Further, the presentation of these substrates to the gut microbiota, as influenced by the relatively understudied host digestive processes occurring in the small intestine, is equally important. Future work could therefore aim to determine which of these pathways are upregulated and downregulated in disease states, such as autism and depression (gut-brain), NAFLD (gut-liver), chronic kidney disease (gut-kidney), and cardiovascular disease (gut-heart). Further, a combination of human- and culture- (in vitro and in vivo) based studies could resolve the spectrum of protein and fat degradation present among healthy individuals, in order to further our understanding of nutrient cycling in gut microbial ecosystems, and thus gain a necessary perspective for improving wellness.

## Additional files

Additional file 1: Pathways of basic amino acid fermentation by the human gut microbiome. Pathways have been simplifed to show major end-products. Where ‘SCFA’ is listed, either acetate, propionate or butyrate can result from catabolism of the substrate. (PDF 181 kb)

Additional file 2: Pathways of aromatic amino acid fermentation by the human gut microbiome. Pathways have been simplified to show major end-products. Where ‘SCFA’ is listed, either acetate, propionate or butyrate can result from catabolism of the substrate. (PDF 174 kb)

## Abbreviations

APC: Antigen presenting cell
BCFA: Branched-chain fatty acid
GABA: 4-Aminobutryate
GI: Gastrointestinal
IBD: Inflammatory bowel disease
IBS: Irritable bowel syndrome
IEC: Intestinal epithelial cell
NAFLD: Non-alcoholic fatty liver disease
SCFA: Short-chain fatty acid
TLR: Toll-like receptor
UC: Ulcerative colitis
