# Supplementary figures and images for: Macronutrient metabolism by the human gut microbiome: major fermentation by-products and their impact on host health
Source: Microbiome. 2019 Jun 13;7:91. doi: 10.1186/s40168-019-0704-8 (PMC6567490; doi:10.1186/s40168-019-0704-8)

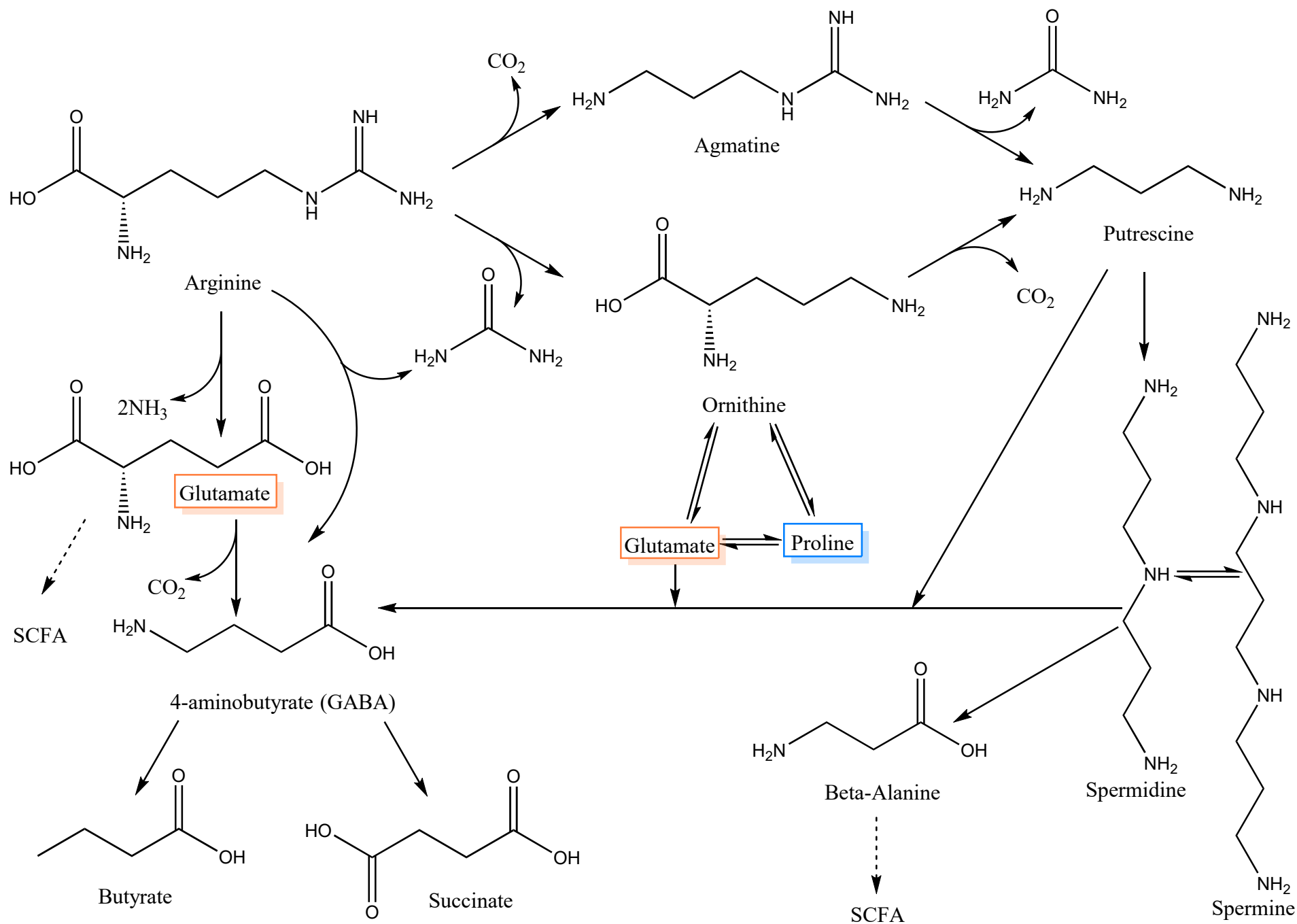

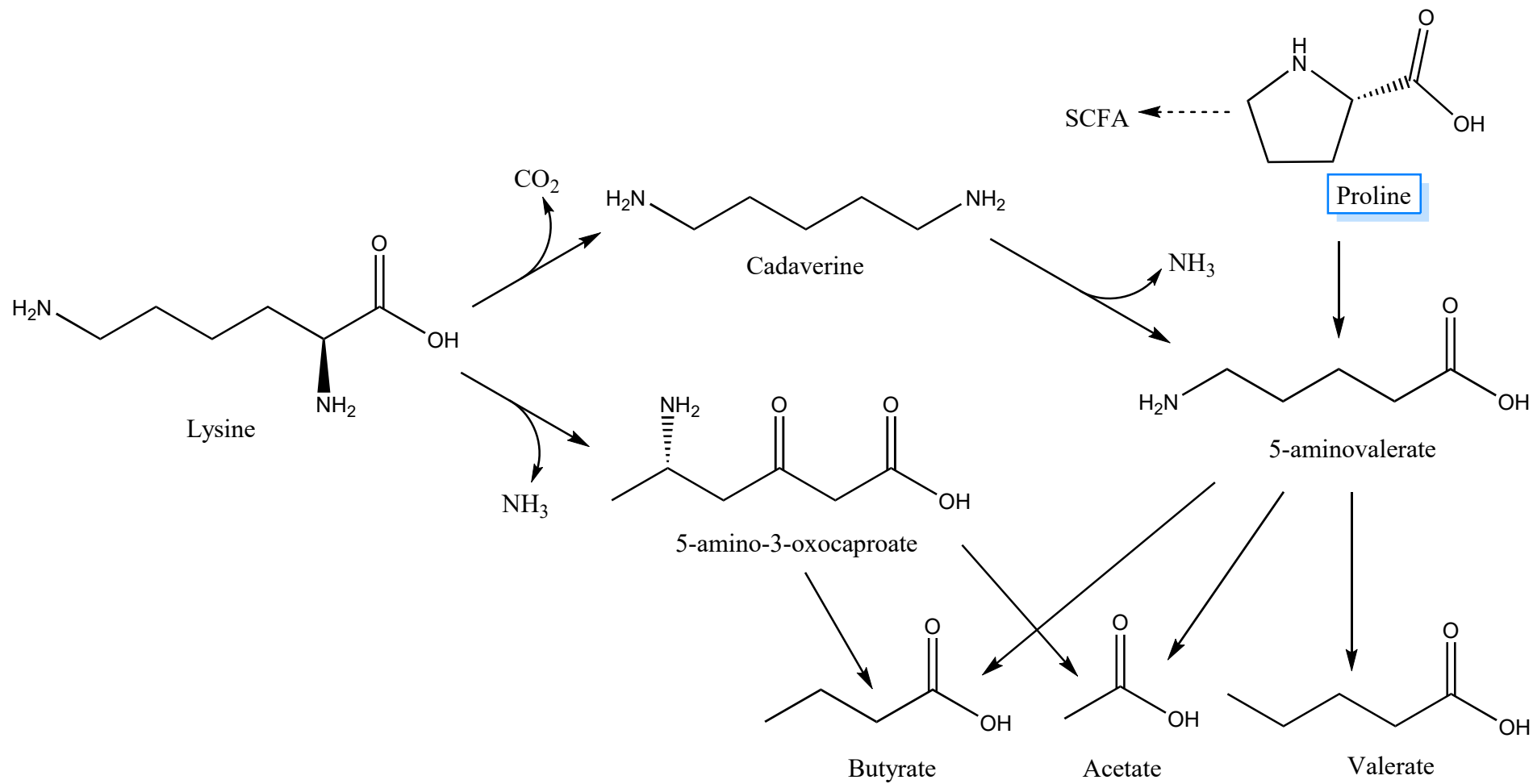

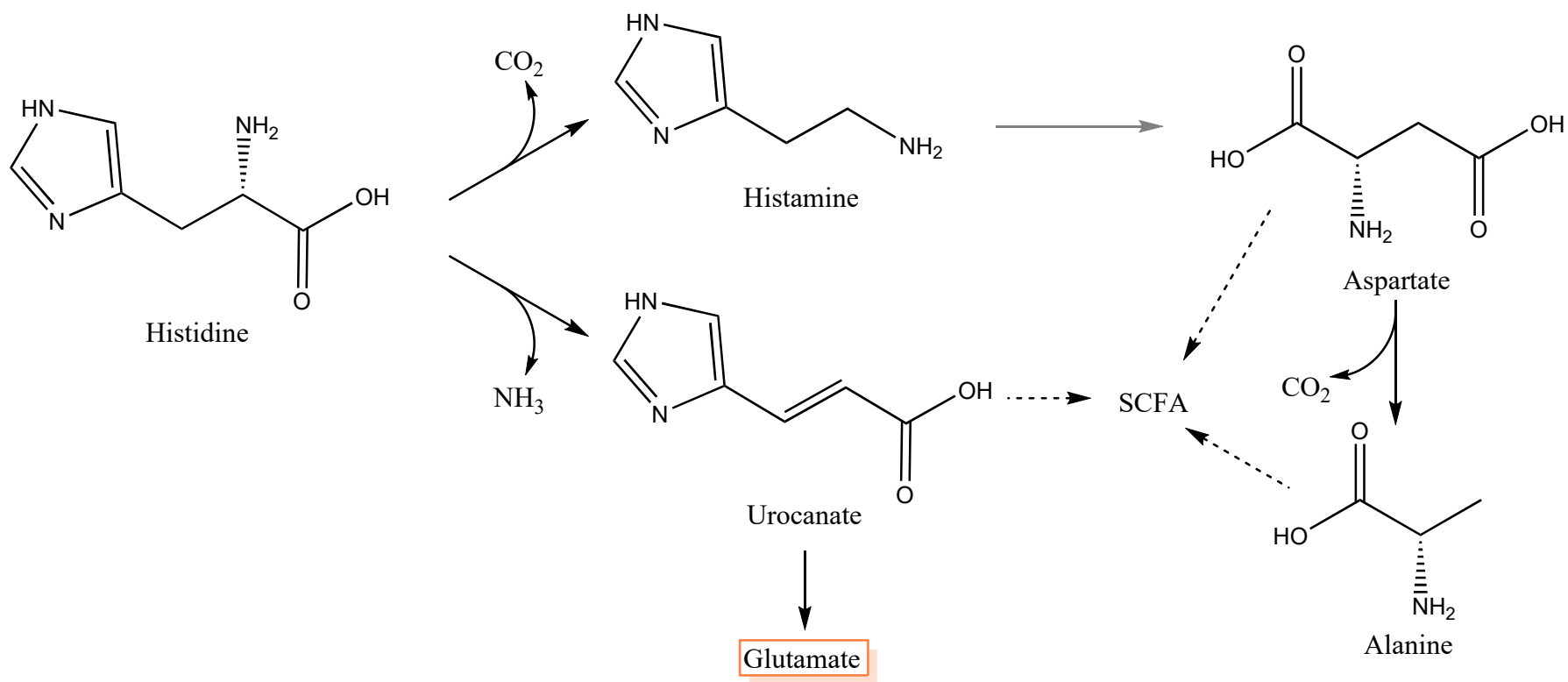

Supplement: Supplementary file 1 — Pathways of basic amino acid fermentation by the human gut microbiome. Pathways have been simplifed to show major end-products. Where ‘SCFA’ is listed, either acetate, propionate or butyrate can result from catabolism of the substrate. (PDF 181 kb) [file 40168_2019_704_MOESM1_ESM.pdf]

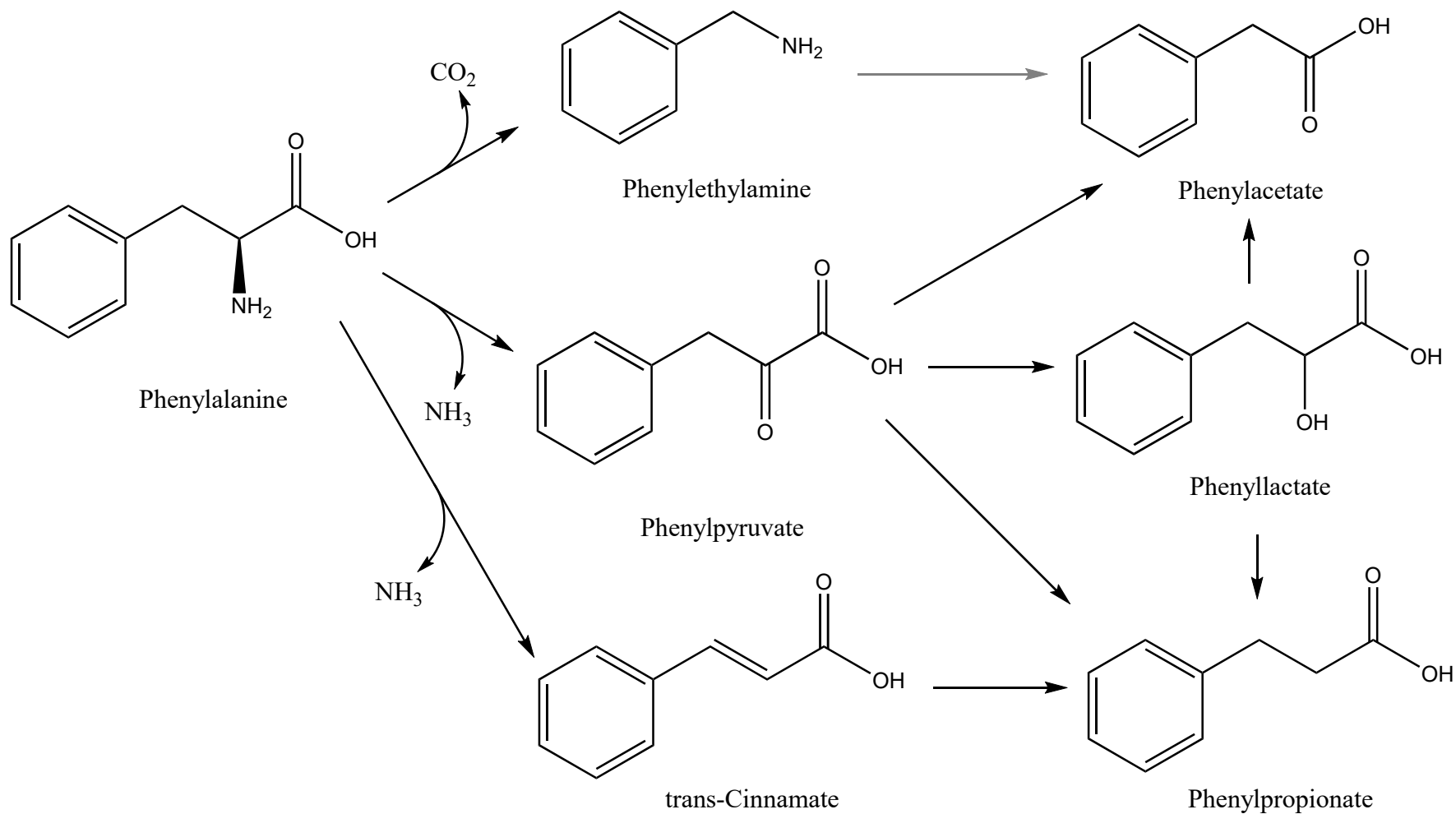

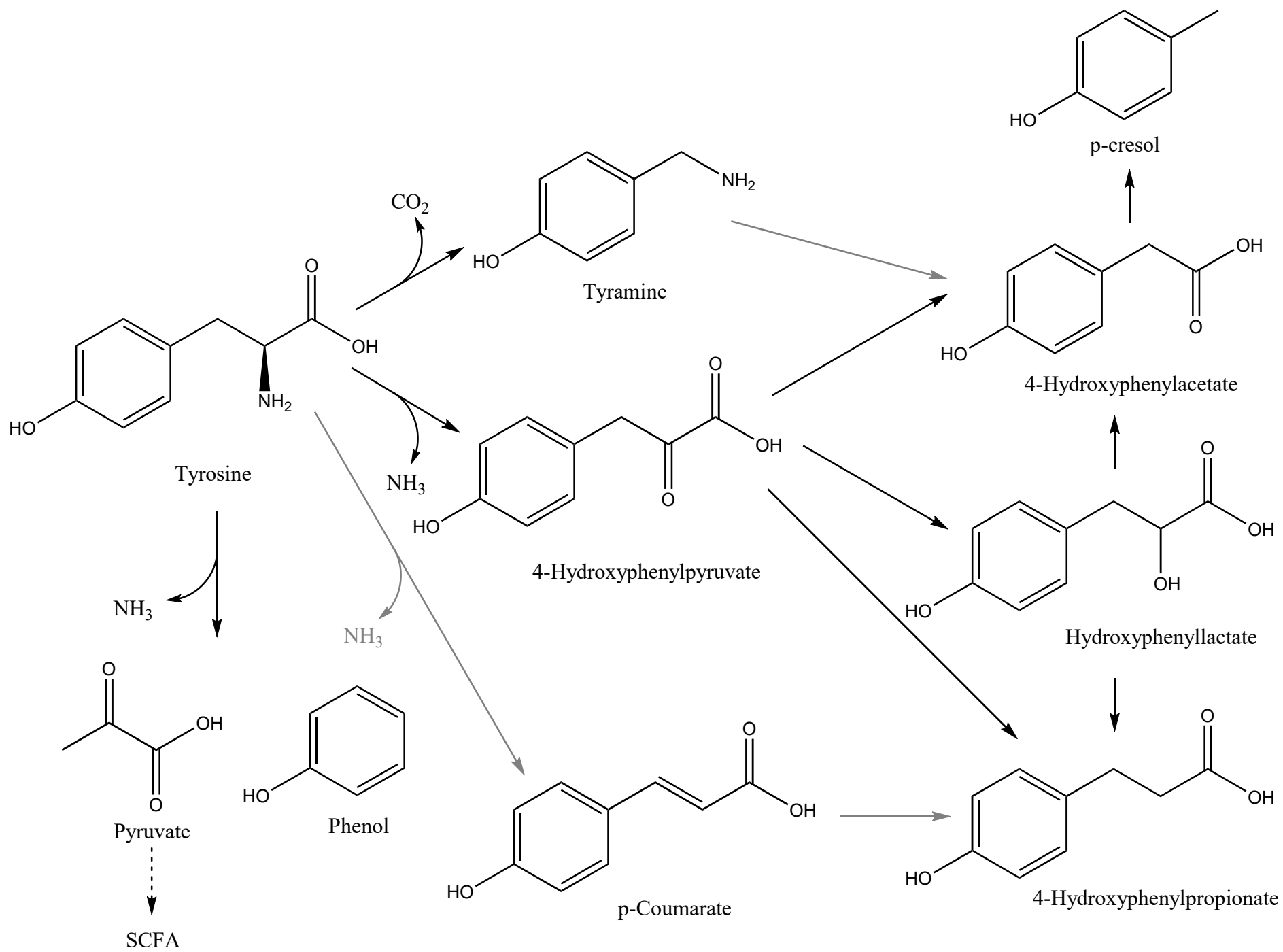

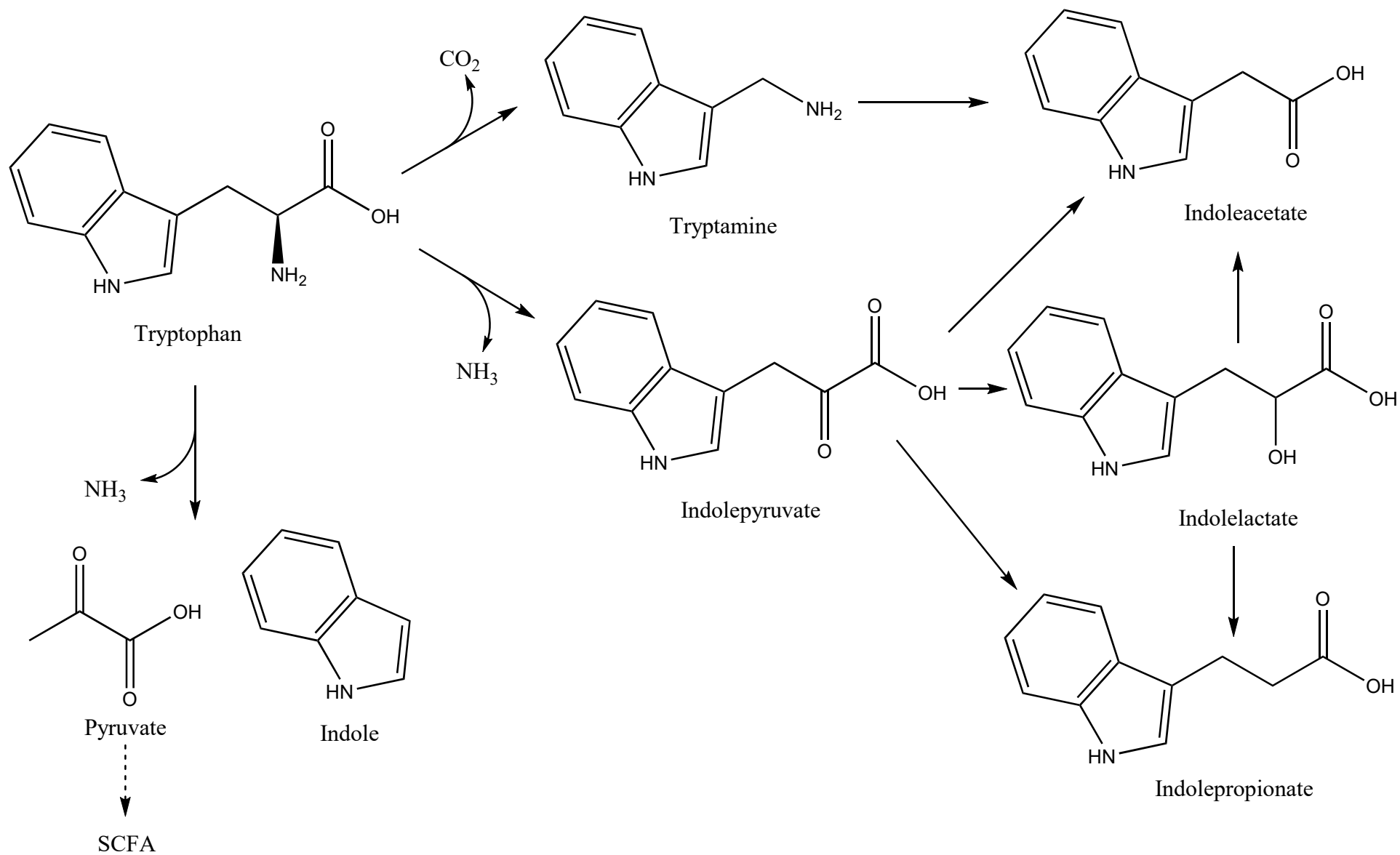

Supplement: Supplementary file 2 — Pathways of aromatic amino acid fermentation by the human gut microbiome. Pathways have been simplified to show major end-products. Where ‘SCFA’ is listed, either acetate, propionate or butyrate can result from catabolism of the substrate. (PDF 174 kb) [file 40168_2019_704_MOESM2_ESM.pdf]
